# Limited Nesting Stress Alters Maternal Behavior and *In Vivo* Intestinal Permeability in Male Wistar Pup Rats

**DOI:** 10.1371/journal.pone.0155037

**Published:** 2016-05-05

**Authors:** Nabila Moussaoui, Muriel Larauche, Mandy Biraud, Jenny Molet, Mulugeta Million, Emeran Mayer, Yvette Taché

**Affiliations:** 1 Center for Neurobiology of Stress, CURE: Digestive Diseases Research Center, Digestive Diseases Division, Department of Medicine and Brain Research Institute, University of California Los Angeles, and VA Greater Los Angeles Healthcare System, Los Angeles, California, 90073, United States of America; 2 Department of Anatomy/Neurobiology, University of California Irvine, Irvine, CA, 92697–4475, United States of America; University of Rennes-1, FRANCE

## Abstract

A few studies indicate that limited nesting stress (LNS) alters maternal behavior and the hypothalamic pituitary adrenal (HPA) axis of dams and offspring in male Sprague Dawley rats. In the present study, we evaluated the impact of LNS on maternal behavior in Wistar rats, and on the HPA axis, glycemia and *in vivo* intestinal permeability of male and female offspring. Intestinal permeability is known to be elevated during the first week postnatally and influenced by glucocorticoids. Dams and neonatal litters were subjected to LNS or normal nesting conditions (control) from days 2 to 10 postnatally. At day 10, blood was collected from pups for determination of glucose and plasma corticosterone by enzyme immunoassay and *in vivo* intestinal permeability by oral gavage of fluorescein isothiocyanate–dextran 4kDa. Dams exposed to LNS compared to control showed an increase in the percentage of time spent building a nest (118%), self-grooming (69%), and putting the pups back to the nest (167%). LNS male and female pups exhibited a reduction of body weight by 5% and 4%, adrenal weights/100g body weight by 17% and 18%, corticosterone plasma levels by 64% and 62% and blood glucose by 11% and 12% respectively compared to same sex control pups. In male LNS pups, intestinal permeability was increased by 2.7-fold while no change was observed in females compared to same sex control. There was no sex difference in any of the parameters in control pups except the body weight. These data indicate that Wistar dams subjected to LNS during the first postnatal week have an altered repertoire of maternal behaviors which affects the development of the HPA axis in both sexes and intestinal barrier function in male offspring.

## Introduction

Early adverse life events are well recognized to have impact on brain development and to contribute to psychiatric illness later in life [1]. Convergent clinical reports also indicate that they predispose to functional gastrointestinal disorders such as irritable bowel syndrome (IBS) [2]. In animals, the early-life stress model most commonly used is the maternal separation paradigm that consists in isolating pups intermittently from the dam usually 3 hours daily during periods ranging from postnatal days (PND) 2 to 14 or 4 to 21 [3–5]. This is achieved preferentially in Wistar rats due to the high sensitivity of this strain to the enduring effects of chronic maternal separation [6]. As adults, pups exposed to intermittent neonatal maternal separation develop a long term hyper responsive hypothalamic-pituitary-adrenal (HPA) axis [4, 5], anxiety and depression-like behaviors [7], and alterations of brain-gut interactions, which resemble characteristic IBS features [8–10]. In particular, adult rats display an impairment of intestinal mucosal barrier integrity [3] and visceral hypersensitivity [11–14].

Others experimental models of neonatal stress such as chronic social stress and limited nesting stress (LNS) have been also described to alter maternal behavior and leading to anxiety and depressive-like behaviors in adult offspring [15, 16]. The LNS procedure consists of placing dams in a cage with restricted nesting and bedding material after parturition starting between PND 1 to 3 for one week [16, 17]. Dams exposed to LNS from PND 2–9 display abnormal fragmented maternal behaviors during this period, and exhibit mild anxiety and alterations of hypothalamic gene expression of corticotropin releasing factor (CRF) when assessed at the end of LNS [18]. The offspring at PND 9 also showed perturbations of the stress system, and when adult, anxiety-like behavior, muscle hyperalgesia and visceral hypersensitivity [18–22]. The postnatal limited nesting and bedding paradigm has been established to have face validity to recapitulate elements of human postnatal conditions where the mother is present but chronically provides erratic or unpredictable maternal care in an impoverished environment [16, 18]. However, the impact of LNS on maternal behavior and HPA axis has been characterized mainly in rats of Sprague Dawley strain and their male pups [17–19] and no data are available in Wistar rat which represent a sensitive strain to stress exposure [6]. Importantly, the consequences of early-life stress on gut function of the pups are still unknown while the two weeks postnatally represent a sensitive period of maturation for the intestinal barrier, a process in which glucocorticoids have been implicated [23, 24]. Previous studies established that intestinal permeability is elevated during the first weeks of life in association with low plasma levels of glucocorticoids in rats [24] and thereafter decreases progressively until adulthood in rodents [24, 25] and human [26]. Glucocorticoids play a major role in the maturation events of various organ systems including the development of the epithelial barrier function by stimulating the morphogenesis of the small intestine and the colon in rats [23, 27, 28]. The elevated intestinal permeability in neonate facilitates the development of food tolerance and immune function by enabling the access of antigens to immune cells of the gut [29]. However, a compromised intestinal barrier function at adulthood can be associated with adverse effects such as bacterial translocation [13, 24] and nerve endings sensitization contributing to visceral hypersensitivity development in rodents and human [30–34].

In the present study, we first investigated the effects of the LNS from PND2 to 10 on maternal behavior in Wistar rats. Then, we evaluated the impact of LNS on the PND10 offspring including components of the HPA axis by assessing changes in the adrenal weight and plasma corticosterone levels. At this stage of development, there is evidence that pups display a hyporesponsive HPA axis [35, 36]. The HPA axis maturation process can be influenced by the state of glycemia. Specifically in the early postnatal period an hypoglycemia exposure could lead to the development of hyper-responsive HPA axis later in life [37]. Therefore we also assessed whether LNS has impact on blood glucose of the pups. Lastly, we examined the integrity of the intestinal epithelial barrier by measuring the state of *in vivo* intestinal permeability in PND10 pups. For comparison, we also included a group of offspring subjected to a brief maternal separation (BMS). This procedure is known to improve resilience and HPA axis response to stress at adulthood [38–40]. Except otherwise stated, all experiments were performed in both male and female pups to examine whether there are sex differences in response to LNS at this early stage.

## Materials and Methods

### Ethic statement

All experimental protocols (#09026–11) were approved by the IACUC Committee of the VA Greater Los Angeles Healthcare System which is under the auspices of the OLAW Assurance of Compliance (A3002-01) in accordance with NIH guidelines.

### Animals

Primiparous–pregnant Wistar rats were shipped on gestational day G-15 from Harlan facilities (San Diego, California, CA, USA) and were immediately housed individually in polypropylene cages under conditions of controlled temperature 23°C±1°C and lighting 12:12 h light/dark cycle (6 pm lights off / 6 am lights on). Food (Prolab RMH 2500; PMI Nutrition International, Inc., Brentwood, MO, USA, USA) and water were available *ad libitum*. The last change of bedding was performed 3 days before parturition. Cages were inspected for the presence of pups at a 12-h interval and the date of birth was labelled as day 0. On PND2, between 9:00 and 10:00 am pups were sexed and litters were mixed and culled to a maximum of 12 pups and minimum of 10 with an equal number in each sex.

### Experimental conditions postnatally

#### Limited nesting stress

The LNS was performed essentially as detailed previously [16, 17]. Briefly, on postnatal day 2 each mother with pups was placed in a standard cage fitted with a wire mesh (McNichols Quality Standard Expanded Metal, Aluminum Type 3003-H14, 3/16. 032 Standard, McNichols, Co. Los Angeles, CA, USA) bottom that was positioned 2.5 cm above the floor to allow passage of excrements. The bedding was reduced to cover the cage floor sparsely and nesting material consisted of half a piece of paper towel (10 x 5 cm) that the dam shred and used to construct a rudimentary nest area. Control groups (CTL) were maintained in standard cages with direct bedding contact, and one paper towel as nesting material. Cages were completely undisturbed (unchanged) from PND 2 to 10.

#### Brief maternal separation procedure

The exposure to BMS was performed as described previously [40]. Housing conditions were similar to those of CTL group except that from PND2 to 9 pups were separated from the dam for 15 min daily between 9:00 and 10:00 am. For each litter, pups were removed altogether from their home cage and kept in temperature controlled cages at 28°C±1°C with normal bedding.

### Experimental design

Male and female pups (5–7 pups/sex/group) taken from one CTL and one LBS litter were weighed and euthanized by decapitation on the morning of PND10. The trunk blood was collected within 15 sec for corticosterone plasma levels determination and both adrenal glands were weighed. In LNS, CTL and BMS groups (4–13 pups/sex/group), intestinal permeability assay was performed on PND between 9:00 am and 1:00 pm and corticosterone plasma levels were measured by collecting blood from the facial vein starting at 1:00 am in lightly hand restrained conscious pups. Lastly, pups from LNS and CTL groups (two litters in each group) were used to assess glucose levels in blood taken from the facial vein under the same conditions described above.

### Assessment of maternal behavior

Each litter was randomly assigned to LNS or CTL groups. The assessment of maternal behavior was performed on 7 dams/group once daily from PND2 to PND9 for a 50 min period starting between 9:00–10:00 am except on PND2 where the assessment was done 1 h after the determination of the sex and the pup counting. Within each observation period, the following dam’s behaviors were monitored continuously by one observer assigned per each dam: licking or grooming any pup (LG), self-licking or grooming (SG), eating or drinking (E), moving involving locomotor activity (M), nest building (NB), carrying/retrieval of pups to put them back to the nest (C) and nursing more than half of the litter (N). Change in nursing postures of the dam was scored as new nursing (AN: another nursing). Dams outside the nest were scored (Out). In addition, the frequency and number of pups outside of the nest were monitored. Frequency and duration of each activity were recorded and cumulative maternal behaviors from PND 2 to 9 was calculated for each component and expressed as the percentage of time spent by the dam in the different activities. Time course of behavioral changes was also calculated.

### Determinations of intestinal permeability, blood glucose and plasma corticosterone in pups on postnatal day 10

#### Intestinal permeability

Total intestinal permeability was measured *in vivo* as detailed in our previous developmental studies in rats [24]. Briefly, rat pups were given an oral gavage (70 μl) of fluorescein isothiocyanate (FITC) labeled 4-kDdextran (FD4), at the dose of 750 mg/kg in 0.9% NaCl (FD4: 4 kDa; Sigma, Milwaukee, WI, USA) at 9:00 am. After 4-h, rats were lightly hand restrained and blood sample (200 μl) was withdrawn within few sec from the facial vein of conscious pup using heparin-coated capillaries (SARSTEDT, Saint James Church Road, NC, USA). Samples were placed immediately on ice and centrifuged. Intestinal permeability to FD4 was determined by measuring plasma FD4 concentration using an automatic Synergy HT multi-detection microplate reader (Ex 485 nm; Em 525 nm, BioTek, Winooski, VT, USA).

#### Blood glucose and plasma corticosterone

Blood samples (200 μl) were collected from the facial vein as described above and blood glucose was measured immediately thereafter using a strip-operated glucometer (One-Touch Ultra; LifeScan, Milpitas, CA, USA). In separate two litters, blood obtained from trunk after decapitation or from facial vein in lightly restrained conscious pups was centrifuged (10 min, 2500 g). Plasma was stored at– 80°C until corticosterone determination performed by enzyme immunoassay (Cayman Chemical, Ann Arbor, MI, USA) according to the manufacturer’s instructions. The sensitivity of the assay was 150pg/ml and interassay variability was 12.5%.

### Statistical analysis

Statistical analyses were performed using GraphPad Prism 4 software (GraphPad; San Diego, CA, USA). Different components of maternal behaviors from PND2-9, and adrenal glands weights, plasma corticosterone and blood glucose of pups at PND10 in LNS and control groups were compared by Student t-test. Maternal behavior between LNS and control group was also analyzed at each day by Multivariate analysis of variance with repeated measures with Pillai test statistics. Correlation analyses between the corticosterone plasma levels and adrenals weights were carried out using the Pearson's correlation coefficient. PND10 *in vivo* intestinal permeability was analyzed by ANOVA and Tukey tests for *post hoc* comparisons. Data were expressed as mean ± SEM and *P* value < 0.05 was considered significant.

## Results

### Maternal behavior assessment

**Fig 1:**
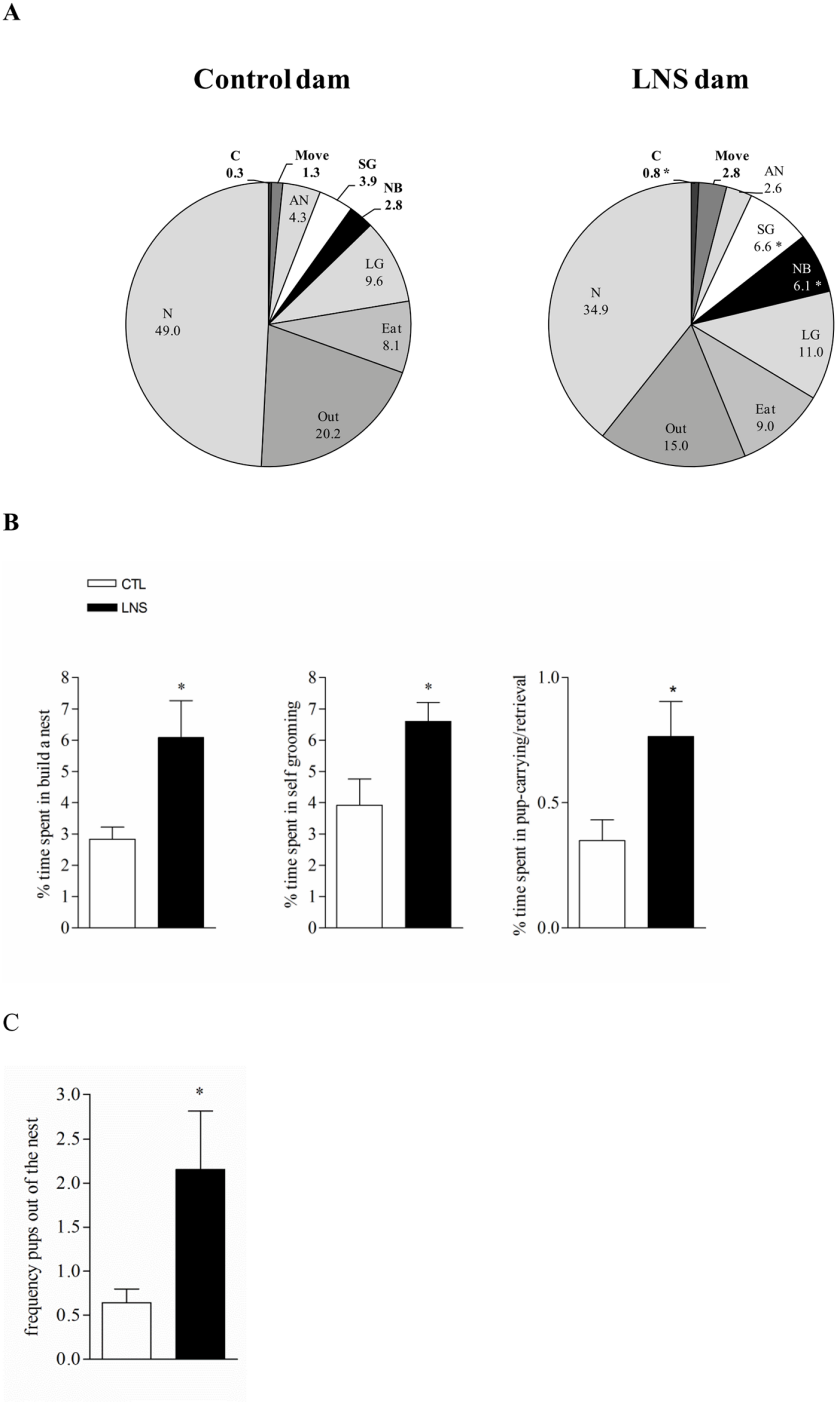
Limited bedding and nesting stress from post-natal days 2 to 9 altered maternal behavior of Wistar rat dams. A: maternal behavior profile in control and LNS (limited nesting stress) dams during the postnatal days (PND) 2–9. Data are expressed as the percentage of time spent by the dams (n = 7) in the different activities measured. Note that “nursing “and “new nursing” activities were measured in 3 dams. (LG: licking and grooming, C: carrying/retrieval: pick up the pups to bring them back to the nest, NB: building a nest, N: nursing, AN: another nursing, SG: self-grooming. B: main behaviors altered in LNS dams from PND 2–9. Data are expressed as the percentage of time spent in the activity ± SEM (n = 7/ group). C: Frequency of pups found out of the nest during PND 2–9. * P<0.05 in LNS vs control (CTL).

In control group, cumulative maternal behaviors assessed from PND 2 to 9 for 50 min in the light phase show that dams spend 49% of their time in nursing and are away from the nest 20.2% of time. Eating, licking and grooming are the two other more prevalent behaviors (Fig 1A). Activities of building a nest and self-grooming represent only 2.8% and 3.9% respectively of the observation period. Dams subject to LNS from PND 2 to 9 show a behavioral profile significantly different from that of control dams as reflected by the higher percentage of time spent building a nest and self-grooming compared to control reaching 118% and 69% increase respectively of that of control dams (Fig 1A, B, n = 7). During the maternal behavior assessment, there is a 3.7 times higher frequency of pups outside of the nest in LNS than in control litters (2.2 ± 0.6 vs. 0.6 ± 0.2; P < 0.05; Fig 1C). Similarly, LNS litters have 3-fold more pups outside of the nest compared to control respectively (3 vs. 1 pup, P < 0.0001, data not shown). LNS dams also show a tendency of moving more (2.8 ± 0.9 vs. 1.3 ± 0.5% compared to control group; p value = 0.19; Fig 1A) and spend significantly more time in carrying/retrieval pups by bringing them back to the limited nest (0.8 ± 0.1 vs. 0.3 ± 0.1% in control; P < 0.05; Fig 1C) while the percentage of time spent licking and grooming the pups and eating/drinking is not different from that of control dams (Fig 1A). Daily analysis of maternal behavior between PND2 to 9 shows that LNS dams spent significantly more time in “carrying/retrieval the pups” at day 8 and in “moving” at day 4 compared to CTL dams. For the all others activities, no significant modifications between LNS and control group were observed (data not shown).

### Body weights

**Fig 2:**
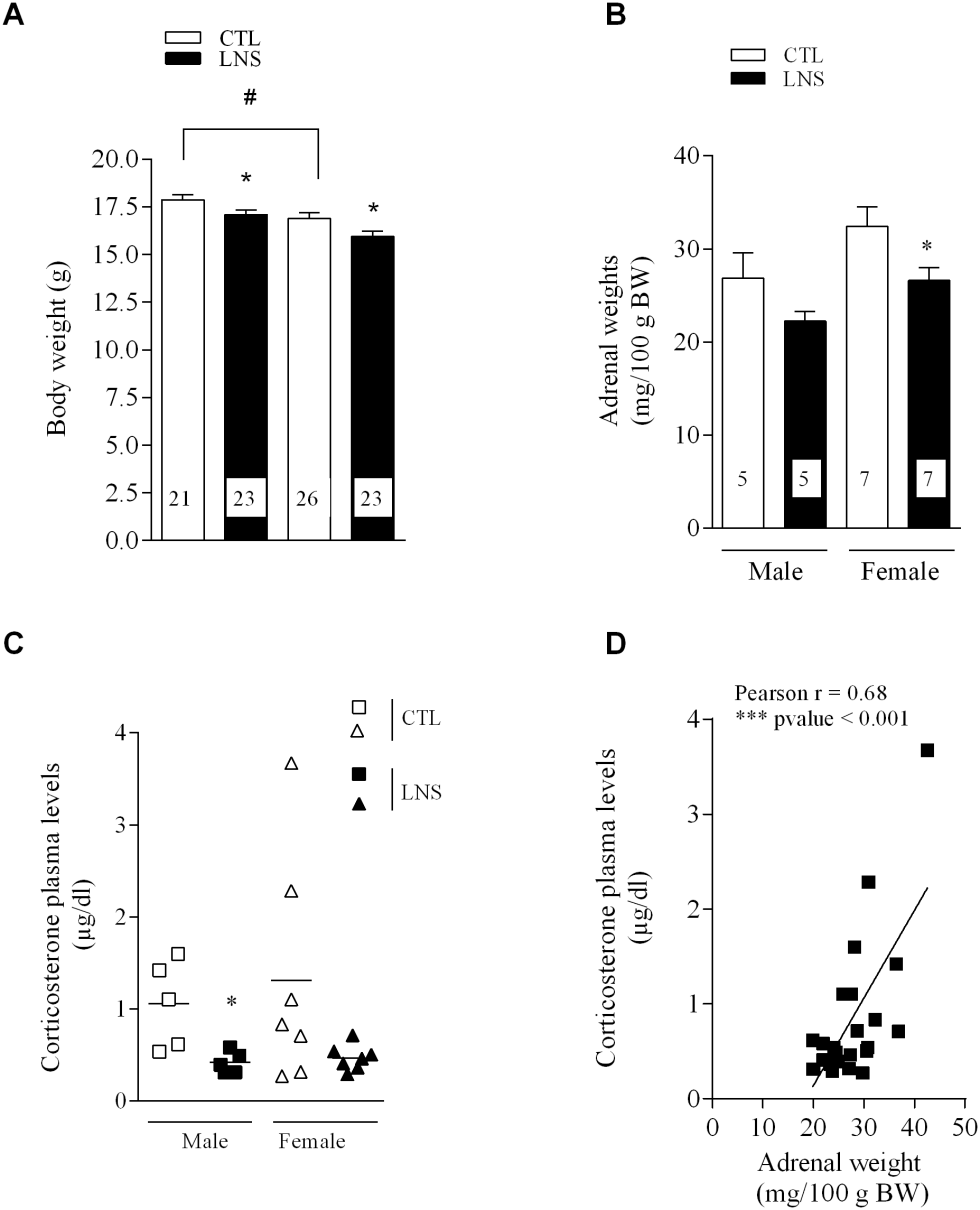
Influence of limited nesting stress (LNS) from post-natal days (PND) 2 to 10 on the body weight and components of the HPA axis in PND 10 pups. A: Body weight of PND10 male and female pups reared under LNS or control (CTL) conditions. Data are expressed as the mean of body weight (g) ± SEM of the number of rats shown at the bottom of each column; *P<0.05 vs CTL pups respectively # P<0.05 vs CLT male pups. B. Adrenal glands weights in male and female pups reared under LNS or CTL conditions. Data are expressed as the mean of adrenal glands weight (mg/100 g BW body weight) ± SEM of number of rats shown at the bottom of each column; P<0.05 vs CTL female pups. C. Corticosterone plasma levels in male and female pups in LNS and CTL groups. Trunk blood was collected after decapitation between 9:00 and 11:00 am. Data are mean ± SEM (5–7 animals per group); *P<0.05 vs male CLT pups. D. Positive correlation between corticosterone plasma levels and adrenals weights in male and female PND10 pups from LNS and CTL groups. (Pearson r = 0.68, ***P < 0.001, n = 24).

Control PND10 male and female pups display a significant difference in body weight with female showing 5% lower values than male pups (16.9 ± 0.3 vs. 17.9 ± 0.3 g, P < 0.05, n = 21–26, Fig 2A). LNS pups at PND10 have a similar (4–5%) reduction of body weight in males (17.1 ± 0.2 vs. 17.9 ± 0.3 g, P < 0.05, n = 21–23) and females (16 ± 0.3 vs. 16.9 ± 0.3 g, P < 0.05, n = 23–26) compared to their respective same sex controls (Fig 2A).

### Adrenal glands weights and plasma corticosterone levels in PND postnatal day 10 pups

In control male and female PND10 pups, the adrenal glands weight/100g BW is similar although there is trend to be higher in females compared to males (Fig 2B). LNS offspring at PND10 shows lower adrenals weights compared to control pups (Fig 2A) with a 17% decrease in males and 18% in females that reaches significance in females (P < 0.05, n = 7) (Fig 2B). LNS pups euthanized at PND10 also have lower corticosterone plasma levels compared to control pups with a significant decrease of 64% in males (0.4 ± 0.1 vs. 1.1 ± 0.2 μg/dl compared to control group, P < 0.05, n = 5) and 62% in females (0.5 ± 0.05 vs. 1.3 ± 0.4 μg/dl, P = 0.0987, n = 7) (Fig 2C). A significant positive correlation is observed between the corticosterone plasma levels and the adrenal weights in PND10 male and female pups (r = 0.68, *P < 0.001, n = 24) (Fig 2D). In another litter of LNS, BMS and CTL male pups, used for a long term study, blood was obtained from facial vein at PND 10 and plasma determinations confirm the significant decrease of corticosterone levels in LNS group while there is no change in BMS pups compared to control group (LNS: 0.6 ± 0.07*, n = 6, BMS: 1.07 ± 0.03, n = 4, *vs*. control: 1.05 ± 0.14 μg/dl, n = 6; *P <0.05 vs LNS).

### Blood glucose level in postnatal day 10 pups

**Fig 3:**
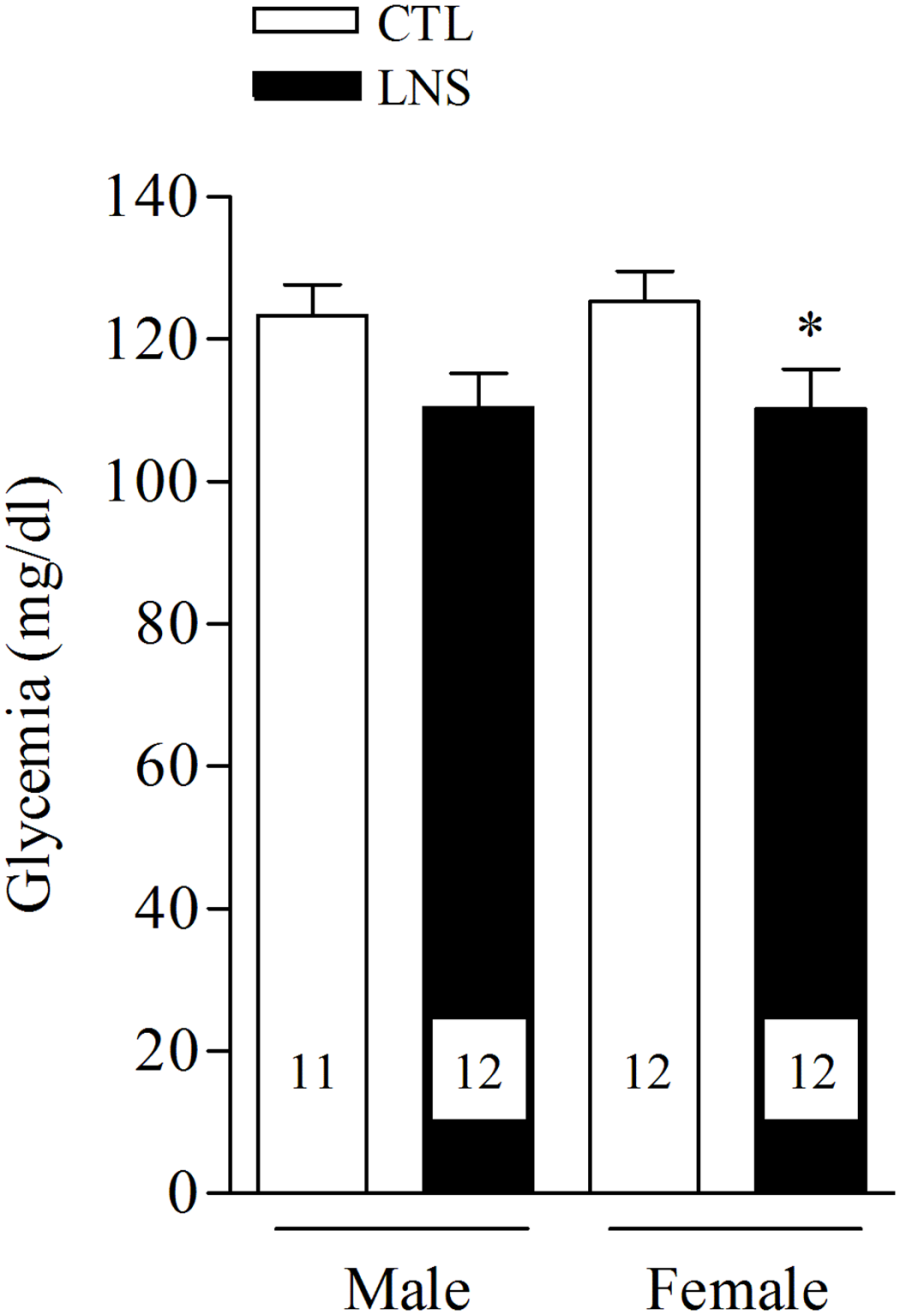
Decreased blood glucose levels in 10 days old male and female pups raised by dams with limited bedding and nesting environment. LNS = Limited bedding and nesting, CTL = control. Data are expressed as the mean ± SEM. The number of rats is shown at the bottom of each column; *P<0.05 vs male CTL pups.

The blood glucose values in male and female control pups are not significantly different. Exposure to LNS reduces blood glucose in PND10 pups similarly in males and females (11% and 12% respectively) with a significant effect in LNS female pups (P <0.05, n = 11−12/group; Fig 3).

### In vivo intestinal permeability in postnatal day 10 pups

**Fig 4:**
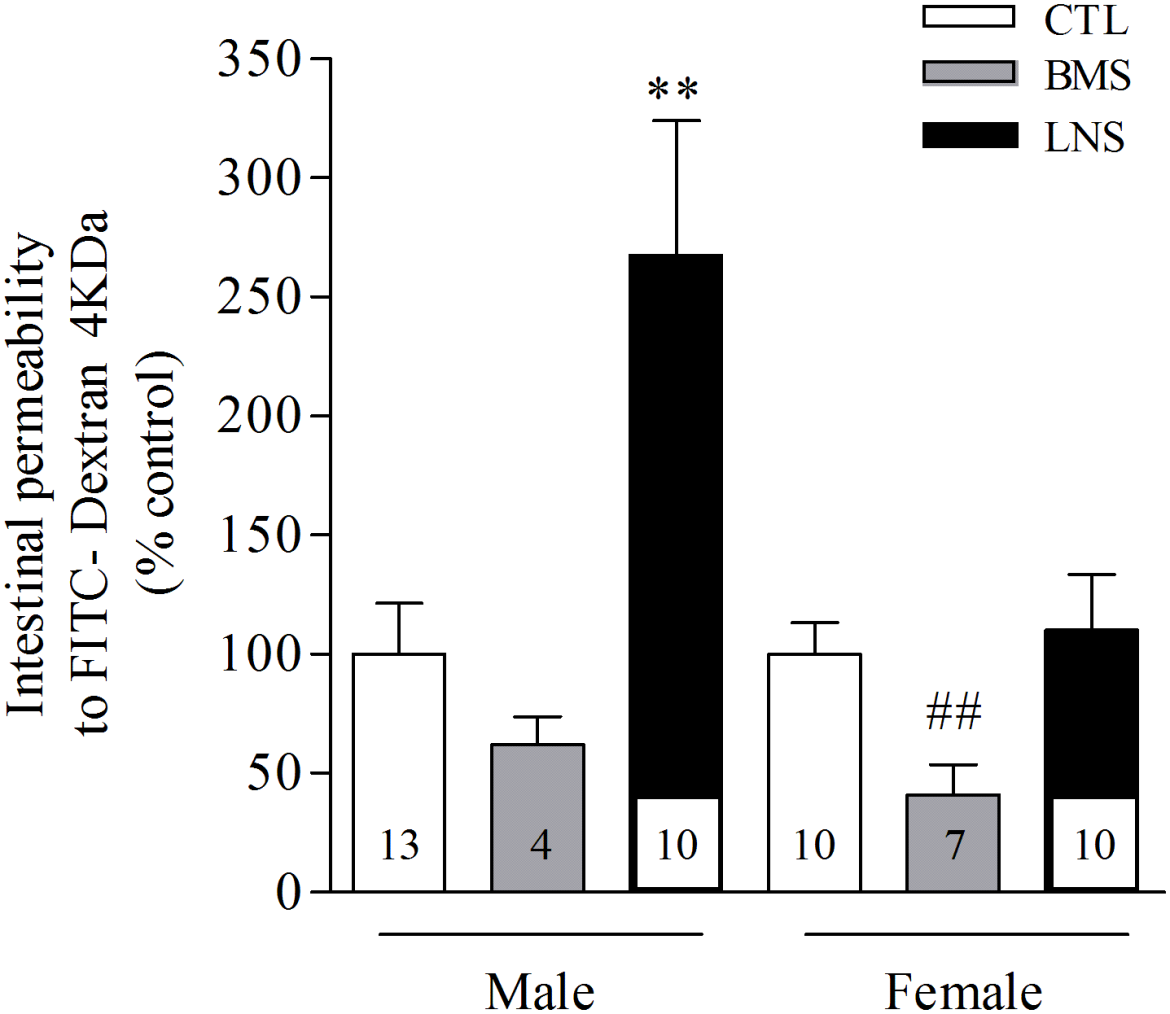
The *in vivo* intestinal permeability to FITC—Dextran 4KDa (FD4) is increased in 10 days old male pups raised by dams with limited bedding and nesting environment and decreased in female pups exposed to brief maternal separation from postnatal days 2 to 10 LNS = Limited bedding and nesting stress, BMS = brief maternal separation, CTL = control. Data are expressed as the mean ± SEM of the number of rats shown at the bottom of each column; **P <0.01 vs male CTL pups (Tukey post hoc test); ##P <0.01 vs female CTL pups.

PND10 control pups do not show significant sex difference in intestinal permeability to FD4 monitored in the plasma 4 h after oral gavage (CTL males: 62.6 ± 14.6 μg/ml, n = 13, vs. CTL females: 76.4 ± 19.4 μg/ml, n = 10). Limited nesting stress alters significantly the intestinal permeability to FD4 in PND10 male pups resulting in a 167% increase compared to control male pups (P <0.001, n = 10–13; Fig 4). By contrast, LNS female pups do not show any modification of intestinal permeability to FD4 compared to the control female group (n = 10). Interestingly, BMS induces a 38% and 63% diminution of intestinal permeability in both male and female pups respectively compared to their respective control groups, with values reaching significance in female pups (P <0.01, n = 4–7; Fig 4).

## Discussion

Our study provides new evidence that the chronic early-life stress model based on limiting the ability of the dam to build a nest for the litter during the first neonatal week alters the maternal behavior of Wistar rats strain and the HPA axis and glycemia similarly in male and female pups and the intestinal epithelial barrier of pups in a sex dependent manner.

Under conditions of limited bedding and nesting material, analysis of cumulative maternal behavior assessed during the light phase for 50 min daily from PND2 to 9 showed that there was an increase in the frequency of pups being out of the nest compared to controls and Wistar dams spent significantly more time in building the nest, self-grooming, bringing back pups to the nest and showed a tendency to move more than the controls. However the time spent in licking/grooming the pups and eating/drinking was not modified compared to control dams. The two previous studies assessing behaviors of dams exposed to LNS performed in Sprague-Dawley rat strain have established alterations in maternal behavior reflecting fragmentation of nurturing and unpredictability while the overall duration of relevant maternal behaviors related to leaking, grooming or nursing were not modified when monitored during the light phase [16, 18]. The present 50 min sampling during the light phase provided evidence that Wistar rat strain also displayed an abnormal pattern of maternal behavior toward offspring created by a deficient and poor rearing environment.

Whether alterations of the physiological development of LNS offspring maybe also contributing to changes in maternal care cannot be ruled out. It is also to note that a more extended behavioral monitoring including the dark phase would also be needed to provide a comprehensive assessment of the LNS impact on the behavior of Wistar dams. In addition, shipping during the gestation of the dams at G15 need to be also taken in consideration as females have to acclimatize to the new environment during the last week of gestation and this can represent an additional stress factor for the dams and the fetus in both CTL and LBS groups.

Next, we assessed the impact of these maternal behavioral changes on components of the HPA axis in Wistar pups. The two first weeks of life in rodents are characterized by low circulating levels of corticosterone, stress-hypo-responsiveness, with the maturation of the HPA axis being highly influenced by the maternal contact [35, 36, 41–44]. In the current study, we showed that PND10 male and female pups reared by dams with limited bedding and nesting had similar 17–18% reduction of the adrenal glands weight and 62–64% lower basal corticosterone plasma levels compared to same sex control pups and there was a positive correlation between corticosterone plasma levels and adrenal weights. The low values of plasma corticosterone in LNS pups were similar to those reported in normal rat pups at PND 3 or 7 which increased few days later to values similar to those we also observed in control pups [44]. Other studies, consistent with our findings, showed also basal corticosterone levels below those of controls in pups PND5-7 associated with reduction of hypothalamic CRF mRNA levels in response to deprivation of maternal care [45]. Of note, 10 days after returning the litter to normal bedding conditions, the LNS pups displayed elevated basal corticosterone plasma levels compared to control pups (Moussaoui et al. unpublished observations). Collectively these data are consistent with a delay in the maturation of the HPA axis in pups reared under conditions of LNS [24, 46]. However, previous reports in PND9 offspring exposed to LNS from PND2 to 9 showed either no change in basal plasma corticosterone levels [17] or an increased level associated with hypertrophy of adrenals [18, 19, 47]. These contrasting results may reflect a strain difference between Wistar (current study) and Sprague Dawley (previous studies) as observed in other physiological parameters in response to chronic changes in the environment [48]. Taken together these findings are indicative that altered maternal behavior of dams during the first week postnatal impacts on circulating levels of corticosterone not only at the end of LNS period but has persistent effects in the form of enhanced basal HPA axis activity. By contrast, brief maternal separation exposure did not modify basal corticosterone plasma levels in PND10 male pups. Other studies showed that Wistar pups exposed to BMS have a normal basal corticosterone plasma levels and a reduction of adrenal responsiveness to psychological or social stress in adulthood [49]. The beneficial effects observed in adult rats in term of stress endocrine response were linked to a modification of maternal behavior after the brief neonatal maternal separation where the dam spent more time in licking and grooming and arched back nursing posture [38].

During the two first weeks of life, newborn rats show a physiologically elevated blood glucose concentration [50, 51] as we observed here in PND10 control male and female. In the present study, PND10 pups exposed to limited bedding and nesting stress displayed a reduction of blood glucose with similar levels than observed in PND7 pups [51]. The low glycemia in LNS pups may be related to the longer time they spent outside the nest compared to controls group and could be consequently less fed leading to a drop of blood glucose.

Glucocorticoids are described to play a major role in the intestinal epithelial maturation process [23, 27, 28] especially during the two first weeks of life [23, 24, 52]. Exogenous administration of glucocorticoids can promote the intestinal maturation by stimulating the morphogenesis of the colon in an age dependent manner with the days 8 to 10 postnatally being the most sensitive period to glucocorticoids [23]. Furthermore, the expression of the tight junction proteins which contribute to the reinforcement of the epithelial barrier are also modulated by glucocorticoids especially claudin proteins which were found to be up regulated by hydrocortisone in an immature human enterocytes model [53]. In the present study, the reduction of adrenals glands weight associated to a lower corticosterone release observed in PND10 male pups could contribute to the delay of the epithelial barrier maturation leading to the significant increase of intestinal permeability to FD4 that we observed here. The expression analysis of intestinal mucosa tight junction proteins in LNS PND10 male pups warrants further investigations to understand whether a lack of tight junction expression is responsible for the elevated intestinal permeability linked with the limited nesting stress. However, important factors such as maternal stimulus, licking/grooming, endocrine and nutritional factors present in breast milk need to be taken into consideration in the maturation process of the epithelium [54–56]. These factors may potentially play an important role in the sex difference observed in intestinal permeability to FD4 where LNS females do not show any modification of intestinal permeability at PND10 while having similar low circulating corticosterone levels. In contrast to LNS, BMS was associated with a significant decrease of intestinal permeability to FD4 in PND10 male and female pups. As the diminution of permeability is age dependent [24, 25] and is the consequence of the gut closure process, the low intestinal permeability in BMS pups could reveal an acceleration of the maturation of the epithelial barrier function.

The most important sex difference effect observed in this study was the response of intestinal permeability to the limited bedding and nesting stress where only male pups were affected. A relationship between compromised epithelial barrier function and visceral pain has previously been demonstrated [30–34]. Of relevance are recent reports showing that LNS from PND 2–9 impacts the visceral hypersensitivity in male but not in female rats when they are adult [21, 22]. An important point to mention is that sex difference observed in LNS model is not occurring in pups with repeated brief maternal separation which highlights the importance of the nature of the stressor and its duration, either repeated intermittent of short duration or chronic when applied during postnatal development.

## Conclusions

In summary, we have obtained new evidence that Wistar rats represent a sensitive strain to study the impact of limited bedding and nesting stress on brain-gut interactions. LNS dams responded by an abnormal maternal behavior impacting the HPA axis and intestinal epithelial barrier in the offspring. At PND10, pups presented a diminution of adrenal glands weight with a reduction of plasma corticosterone and blood glucose. LNS male pups displayed also a leaky gut by showing an abnormally elevated *in vivo* intestinal permeability. When viewed together, these data suggest that PND10 pups raised by dams with limited nesting have a delayed maturation of the HPA axis and of epithelial intestinal barrier specifically in male pups. A possible causal relationship between these early findings and the development of visceral hypersensitivity reported in adult male unlike female Wistar rats [21, 22] needs to be explored as well the possible implications in other diseases namely the development of regressive autism for which a role of increased intestinal barrier permeability and microbiota has been suggested during the first months postnatally [57].
